# Effects of a High-Sodium, Carbohydrate-Free Multi-Electrolyte Supplement on 15 km Treadmill Time-Trial Performance in Warm and Humid Conditions: A Randomized, Single-Blind, Placebo-Controlled Crossover Trial

**DOI:** 10.3390/sports14100435

**Published:** 2026-10-07

**Authors:** Philip Prins, Jeffrey D. Buxton, Abigail Helsel, Sarah Bonnett, Luke DeMember, Jackson Timko, Timothy Noakes

**Affiliations:** 1 Department of Exercise Science, Grove City College, Grove City, PA 16127, USA; 2 Emeritus Faculty of Health Sciences, University of Cape Town, Cape Town 7925, South Africa

**Keywords:** sodium, fluid balance, endurance performance, heat stress, thermoregulation, running time trial

## Abstract

Sodium ingestion may support fluid balance and endurance performance in the heat, but most previous studies have used sodium-loading, hyperhydration, or carbohydrate–electrolyte protocols. This study examined the effects of a high-sodium, carbohydrate-free multi-electrolyte supplement (HSES) on 15 km treadmill time-trial performance and physiological responses in trained male runners under warm-humid conditions. In a randomized, single-blind, counterbalanced crossover design, ten trained male runners (V̇O_2_max: 57.3 ± 8.1 mL·kg^−1^·min^−1^) completed two 15 km treadmill time trials at 26.7 °C and 60% relative humidity. Participants received HSES or a flavor-matched placebo (PLA) 30 min before exercise and every 15 min of running time. Repeated-measures analysis of cumulative completion time showed a significant main effect of condition (*p* = 0.034) and a significant condition × distance interaction (*p* = 0.023). Segment pace also showed a significant condition × segment interaction (*p* = 0.001); participants ran faster during HSES over the opening 0–5 km (*p* < 0.001), pace did not differ during 5–10 km (*p* = 0.091), and participants ran faster during PLA over the final 10–15 km (*p* = 0.032). Mean final 15 km completion time was 68.76 ± 12.28 min during HSES and 70.18 ± 13.33 min during PLA, a mean difference of 1.42 min (~2.0%). However, the direct paired comparison of final completion time did not reach statistical significance (HSES − PLA: −1.42 min; 95% CI, −3.60 to 0.77 min; *p* = 0.176; Cohen’s *dz* = −0.46). No significant between-condition differences were observed in measured hydration-related, cardiovascular, metabolic, or perceptual outcomes. Estimated core temperature was higher during HSES at 15 km and 10 min post-exercise. Pacing differed between conditions during the 15 km time trial, with a faster opening 5 km during HSES and a faster final 5 km during PLA. Final 15 km completion time was approximately 2.0% faster during HSES, although the difference was not statistically significant.

## 1. Introduction

Prolonged endurance exercise in hot and humid environments imposes substantial cardiovascular, thermoregulatory, and fluid-regulatory strain. As metabolic heat production rises, the maintenance of exercise intensity requires simultaneous support of active skeletal muscle blood flow and heat dissipation through increased skin blood flow and sweating [1,2,3,4]. Progressive body water loss can reduce plasma volume, contribute to cardiovascular drift, and increase the physiological strain associated with prolonged exercise in the heat [5,6,7]. These responses are particularly relevant during distance running because sweat rates commonly exceed 1–2 L·h^−1^, while sweat sodium concentration varies widely among athletes, producing large interindividual differences in total sodium losses [8,9].

Sodium intake before or during exercise may help preserve fluid balance by promoting fluid retention and supporting extracellular fluid volume, plasma osmolality, and thirst regulation [10,11]. Because sodium is the primary extracellular electrolyte lost in sweat, sodium-containing fluids have often been proposed as a strategy to reduce the fluid-regulatory and cardiovascular consequences of prolonged exercise in the heat.

However, sodium replacement should not be viewed simply as matching sweat sodium loss or as justification for aggressive fluid intake, as work by Noakes and colleagues on exercise-associated hyponatremia (EAH) emphasizes that sodium balance must be interpreted in relation to total fluid balance and body mass change [12,13,14]. Likewise, changes in serum sodium concentration during endurance exercise reflect the interaction between fluid intake, body water loss, sweat sodium loss, and sodium ingestion rather than sodium loss alone [8]. Thus, the potential performance benefit of sodium supplementation is most likely to occur when sodium improves fluid retention, supports plasma volume, or helps maintain cardiovascular function during prolonged heat stress without promoting overhydration.

Only a few controlled studies have examined high-sodium or sodium-induced hyperhydration strategies using performance-based outcomes in the heat. Hamouti et al. [15] reported that sodium plus water ingested before 120 min of cycling in the heat better preserved plasma volume, cardiac output, and stroke volume and increased mean work rate during the subsequent cycling time trial, despite no clear improvement in heat dissipation. More recently, Convit et al. [16] found that sodium hyperhydration improved cycling time-trial performance in female cyclists exercising in hot and humid conditions without significant changes in rectal temperature, heart rate, thermal sensation, or perceived exertion. In contrast, Gigou et al. [17] reported that sodium-induced hyperhydration reduced body mass loss, heart rate, and rectal temperature during an 18 km treadmill running time trial in warm conditions but did not improve performance. Together, these studies suggest that sodium-induced fluid retention may improve performance in some cycling models, yet the translation to running remains uncertain.

This uncertainty is important because running presents a unique performance context. Unlike cycling, any increase in retained fluid also increases the body mass that must be carried during exercise, which may offset some of the potential cardiovascular or fluid-regulatory benefit of hyperhydration [17]. In addition, many commercially available electrolyte products are consumed during exercise rather than as aggressive pre-exercise hyperhydration protocols, and their sodium content may be delivered without carbohydrate. Traditional carbohydrate–electrolyte solutions provide fluid, modest sodium, and exogenous carbohydrate simultaneously, making it difficult to isolate the independent contribution of electrolyte-focused supplementation [18,19]. A high-sodium, carbohydrate-free multi-electrolyte supplement therefore provides a practical model for examining whether an electrolyte-focused beverage can influence running performance in the heat without exogenous carbohydrate provision.

Therefore, the purpose of this study was to examine the effects of a high-sodium, carbohydrate-free multi-electrolyte supplement compared with a flavor-matched placebo on 15 km treadmill time-trial performance, thermoregulation, metabolic responses, hydration status, and perceptual responses in trained male runners exercising in warm and humid conditions. We hypothesized that HSES would improve 15 km time-trial performance compared with the placebo. A secondary aim was to determine whether any performance effect was accompanied by measurable differences in hydration status, substrate metabolism, thermoregulation, or perceptual responses.

## 2. Materials and Methods

### 2.1. Experimental Design

A randomized, single-blind, counterbalanced crossover study was conducted to evaluate the effects of a high-sodium, carbohydrate-free multi-electrolyte supplement (HSES) compared with a flavor-matched placebo (PLA) on endurance performance, metabolic and thermoregulatory responses, hydration status, and perceptual responses during a 15 km treadmill time trial in 10 trained male endurance runners. Each participant completed three total laboratory sessions. Session 1 included informed consent, health screening, familiarization with the experimental procedures and perceptual scales, standard anthropometric assessments, body composition measurement, and a graded maximal exercise test on a treadmill to determine maximal oxygen uptake (V̇O_2_max). Sessions 2 and 3 involved the experimental trials, separated by a 7-day washout period to allow recovery and minimize potential carryover effects. Participants arrived in a fasted state and received either HSES or PLA 30 min prior to exercise and every 15 min during the time trial. Experimental trials were performed in an environmental chamber maintained at 26.7 °C and 60% relative humidity (RH). Blood metabolites, including glucose and lactate, were measured via finger prick before beverage ingestion, following the pre-exercise heat exposure period, and at 5 km intervals throughout exercise. Perceptual measures, including rating of perceived exertion (RPE), affect, thermal sensation, thirst, and gastrointestinal distress, were also assessed at 5 km intervals. Heart rate and respiratory gas exchange were continuously monitored during exercise using wearable heart rate monitoring and indirect calorimetry, respectively. Estimated core body temperature was monitored continuously using a wearable sensor. Hydration status was assessed via urine specific gravity and body mass before and after exercise. All procedures were approved by the Grove City College Institutional Review Board (IRB number 128–2025), and all participants provided written informed consent prior to participation. The study was retrospectively registered with the ISRCTN registry (ISRCTN39215157) on 24 September 2026. All testing was conducted in the Grove City College Exercise Science Department Human Performance Environmental Physiology Laboratory.

### 2.2. Participants

A single cohort of 10 trained male endurance runners was recruited from local running communities and competitive racing groups (Table 1). Inclusion criteria required that participants: (1) were male and between 18 and 50 years of age; (2) ran a minimum of 40 km per week; (3) had trained for or completed a 10 km run within the previous three months; (4) completed a 10 km run in under 60 min; (5) had a minimum of two years of distance running experience; (6) demonstrated a maximal oxygen uptake (V̇O_2_max) ≥ 50 mL·kg^−1^·min^−1^; and (7) reported consuming a habitual mixed diet and were not following a carbohydrate-restricted or other specialized dietary pattern. These inclusion criteria were selected to ensure that participants were sufficiently trained to complete a 15 km treadmill time trial under warm and humid environmental conditions. Participants were excluded if they: (1) had a history of smoking; (2) had known metabolic, cardiovascular, or respiratory disease, including diabetes or chronic electrolyte imbalance; (3) had any orthopedic, neurological, or musculoskeletal condition that contraindicated exercise testing; (4) had a history of exertional heat stroke, recurrent heat exhaustion, or unexplained exertional syncope or intolerance in the heat; or (5) had a history of exercise-associated hyponatremia, syndrome of inappropriate antidiuretic hormone secretion, chronic kidney disease, heart failure, liver failure, or known salt-wasting disorders. Participants were instructed to refrain from vigorous exercise for 24 h prior to each trial, avoid caffeine and alcohol for 48 h, and fast for at least 8 h prior to testing. Before participation, all participants were informed of the study procedures, risks, and potential discomforts and provided written informed consent. Eligibility criteria were verified through health history screening and a graded maximal exercise test.

### 2.3. Pretrial Preparation

Participants were instructed to maintain their usual training frequency and intensity throughout the study without altering their training load. Each participant was provided with a training log and instructed to record the mode, duration, and subjective intensity of each workout throughout the study period. To promote consistency, participants were provided with a copy of their training log from the week prior to the first experimental trial and were instructed to replicate their training pattern as closely as possible before the second experimental trial.

Training intensity was quantified using the session rating of perceived exertion (sRPE) method [20], calculated by multiplying session duration (min) by the RPE reported using the OMNI Walk/Run 0–10 Perceived Exertion Scale [21]. Weekly training load was calculated as the sum of all individual session loads. As shown in Table S1, no statistically significant difference in training load was detected between experimental trials (*p* = 0.355).

Participants recorded all food and beverage intake using the MyFitnessPal mobile application (San Francisco, CA, USA) during the three days preceding each experimental trial. During the familiarization visit, participants received individualized oral and written instructions on how to accurately log dietary intake, including portion sizes and ingredients. Participants were instructed to maintain their habitual dietary pattern and to replicate dietary intake as closely as possible before each experimental trial. Nutrient intake data were used to assess daily energy intake, macronutrient composition, fiber, sugar, and cholesterol. As shown in Table S2, no statistically significant differences were detected between trials for total energy intake (*p* = 0.322), carbohydrate intake (*p* = 0.179), protein intake (*p* = 0.659), or fat intake (*p* = 0.484). Participants were also instructed to refrain from vigorous exercise for 24 h before each trial, avoid caffeine and alcohol for 48 h, and report to the laboratory following an overnight fast of at least 8 h.

### 2.4. Familiarization, Anthropometric Measurements and Maximal Aerobic Capacity

At the first laboratory visit, all experimental procedures were explained to participants. Participants underwent an orientation involving familiarization with the treadmill time-trial protocol, laboratory equipment, measurement instruments, affect measures, and perceived exertion scales. To reduce potential learning effects during experimental sessions, participants were familiarized with all procedures and measurement instruments before completing the graded exercise test.

Following the orientation session, anthropometric measures were obtained, including height, body mass, fat-free mass, fat mass, and percent body fat. Height was measured using a physician’s scale (Detecto, Webb City, MO, USA). Body mass, fat-free mass, fat mass, and percent body fat were assessed using a Tanita bioelectrical impedance analyzer (BIA; MC-980UPlus, Tanita Corporation of America, Arlington Heights, IL, USA).

Finally, V̇O_2_max was assessed via a graded exercise test on a motorized treadmill (Trackmaster TMX425C, Newton, KS, USA) using the Modified Åstrand protocol [22]. Oxygen consumption (V̇O_2_) and carbon dioxide production (V̇CO_2_) were measured continuously using an automated metabolic analyzer system (TrueOne 2400, ParvoMedics, Sandy, UT, USA), which was calibrated before each exercise test using standard reference gases (16% O_2_ and 4% CO_2_). Heart rate was continuously monitored using a chest strap heart rate monitor (Polar H10, Polar Electro, Kempele, Finland). Participants completed a 3 min self-paced warm-up at 0% grade, after which treadmill speed was held constant between 5 and 8 mph and incline increased by 2.5% every 2 min until volitional exhaustion. The highest 30 s average V̇O_2_ value achieved during the test was used to determine V̇O_2_max and verify eligibility for the experimental trials.

### 2.5. Supplementation Protocol

During the second and third laboratory visits, participants received either HSES or PLA 30 min before exercise and every 15 min of accumulated running time during the 15 km treadmill time trial. For the HSES condition, one ready-to-mix HSES serving (Drink LMNT, Inc., Bozeman, MT, USA) contained 1000 mg sodium, 200 mg potassium, and 60 mg magnesium and was mixed in 500 mL of water. Participants consumed the full 500 mL beverage before entering the environmental chamber. During the time trial, participants consumed 250 mL of the assigned beverage every 15 min of accumulated running time until completion, corresponding to one-half of the initial serving volume and providing 500 mg sodium, 100 mg potassium, and 30 mg magnesium per in-trial dose in the HSES condition. All scheduled beverage doses were consumed in full.

Including the pre-exercise dose and all in-trial beverage doses, mean total fluid intake was 1.50 ± 0.17 L during HSES and 1.60 ± 0.17 L during PLA. This corresponded to approximately three total HSES servings and provided 3000 mg sodium, 600 mg potassium, and 180 mg magnesium across the experimental trial. The PLA beverage was provided using the same prescribed fluid volume at each scheduled administration but without added electrolytes. Both beverages were flavored with lemonade-flavored Stur liquid water enhancer (Dyla LLC, Edison, NJ, USA). All beverages were prepared before each experimental trial in opaque sports bottles and were matched as closely as possible for appearance, flavor, and scheduled timing of administration. Because of the high sodium content of HSES, complete taste matching was not possible, and participants may have been able to identify the higher-sodium beverage despite flavor matching, opaque bottles, and presentation of both beverages as having similar ergogenic potential.

### 2.6. Blood Metabolites and Perceptual Measures

Capillary blood samples were collected to assess blood glucose and blood lactate concentrations. Blood glucose was measured using a Keto-Mojo blood glucose monitoring system (Keto-Mojo, Napa, CA, USA), and blood lactate was measured using a Lactate Plus analyzer (Nova Biomedical, Waltham, MA, USA). Measurements were obtained at baseline before beverage ingestion, immediately before exercise following the 30 min passive heat exposure period, at 5 km intervals during the 15 km treadmill time trial, and 10 min post-exercise. All samples were obtained using a single-use lancet after cleansing the fingertip with an alcohol swab and allowing the skin to dry. The first blood droplet was discarded to reduce contamination, and subsequent droplets were used for immediate analysis.

Perceptual responses were assessed throughout each experimental trial, including baseline, pre-exercise, during exercise at each 5 km interval, and 10 min post-exercise. Perceptual measures included rating of perceived exertion (RPE), affect, thermal sensation, thirst, and gastrointestinal (GI) distress. RPE was assessed using the OMNI Walk/Run 0–10 Perceived Exertion Scale [21]. Affective responses were measured using the 11-point Feeling Scale [23], ranging from −5 “very bad” to +5 “very good.” Thermal sensation was assessed using a 9-point scale ranging from −4 “very cold” to +4 “very hot” [24]. Thirst was measured using a 7-point scale ranging from 1 “not thirsty at all” to 7 “very, very thirsty” [25]. GI distress was assessed using a 0–10 severity scale, with 0 indicating no symptoms and 10 indicating severe symptoms [26,27].

### 2.7. Respiratory Gas Exchange and Substrate Oxidation

Respiratory gas exchange was recorded using an automated metabolic analyzer system (TrueOne 2400; ParvoMedics, Sandy, UT, USA). Before each experimental session, the device was calibrated according to the manufacturer’s instructions. Metabolic gases and HR were continuously collected during the TT using a metabolic cart for the assessment of RER, V̇O_2_, V̇CO_2_, V̇E, respiratory rate, HR, and substrate oxidation. Physiological and metabolic variables, including V̇O_2_, V̇CO_2_, V̇E, RER, heart rate, and substrate oxidation, were averaged across each 5 km segment of the 15 km time trial. Fat and carbohydrate oxidation rates were calculated from the nonprotein respiratory quotient using the following stoichiometric equations [28]: fat oxidation (g/min) = (1.67(V̇O_2_)) − (1.67(V̇CO_2_)) and carbohydrate oxidation (g/min) = (4.55(V̇CO_2_)) − (3.21(V̇O_2_)).

### 2.8. Hydration Status and Sweat Rate

Hydration status was assessed from urine specific gravity (USG) and changes in body mass before and after each 15 km time trial. Participants were instructed to arrive at the laboratory in an euhydrated state. A urine sample was collected and immediately analyzed for USG to characterize pre-exercise hydration status, with values ≤1.020 interpreted as consistent with euhydration [7]. USG was measured using a handheld refractometer (Atago PAL-10S, Tokyo, Japan).

Body mass was then measured using a calibrated digital physician scale (Health O Meter^®^ Professional, Pelstar LLC, McCook, IL, USA); participants wore the same dry, minimal clothing for each experimental trial. After completion of the time trial, participants towel-dried, changed into the same dry, minimal clothing, and body mass was reassessed. A post-exercise urine sample was also collected for assessment of USG.

Fluid intake during each trial was recorded from the prescribed beverage volume consumed before and during exercise. Urine output was measured when urine was produced during or after the time trial. Whole-body sweat loss was estimated from the change in body mass corrected for fluid intake and urine output, consistent with prior hydration and exercise–heat stress methods [4,9,29]. Sweat rate was calculated as:

Sweat rate (L·h^−1^) = [fluid intake − urine output + body mass loss] ÷ exercise duration

where body mass loss was calculated as the difference between pre- and post-exercise body mass.

### 2.9. Cardiovascular and Thermoregulatory Measurements

Before each experimental trial, participants were fitted with a heart rate (HR) monitor (Polar H10; Polar Electro, Kempele, Finland) positioned at the xiphoid process. HR was monitored continuously during the 15 km time trial. Blood pressure (BP) was measured using an automated blood pressure monitor (ADView 2 Diagnostic Station, Model 9005BP; American Diagnostic Corporation, Hauppauge, NY, USA) after 5 min of seated rest at baseline, immediately before exercise, and 10 min after completion of the time trial.

Estimated core temperature (Tc) and skin temperature (Tsk) were monitored continuously using a noninvasive wearable temperature sensor (CORE Sensor; greenTEG AG, Rümlang, Switzerland). The sensor was attached to the HR monitor strap and positioned along the left anterior axillary line according to the manufacturer’s recommendations. Temperature data were recorded at baseline, immediately before exercise, at each 5 km interval during the time trial, and 10 min post-exercise. The CORE Sensor estimates Tc using heat flux and Tsk. Similar greenTEG heat-flux sensors have been used during exercise in the heat and have demonstrated acceptable validity and reproducibility compared with gastrointestinal temperature [30]. Because Tc was assessed using a noninvasive heat-flux sensor rather than rectal thermometry, esophageal temperature, or ingestible telemetry, values are reported as estimated Tc throughout the manuscript.

### 2.10. Protocol for 15 km Running Time Trials

All time trials were performed on a calibrated motorized treadmill (TMX425C; Trackmaster, Newton, KS, USA) inside a climate-controlled environmental chamber (Cantrol, Montreal, QC, Canada) maintained at 26.7 °C and 60% RH to model a controlled warm-humid running environment. Airflow was standardized across trials using fixed fan settings. The treadmill time-trial procedures were modeled after prior laboratory-based running performance protocols in which participants completed a fixed distance as quickly as possible while self-selecting pace and adjusting treadmill speed throughout the trial [17,31,32].

Participants reported to the laboratory following an overnight fast of at least 8 h and after refraining from vigorous exercise for 24 h and caffeine and alcohol for 48 h. Upon arrival, participants voided their bladder, provided a urine sample, and were weighed while wearing the same dry, minimal clothing for each trial. Following 5 min of seated rest, baseline measures were obtained, including blood pressure, blood metabolites, thermoregulatory variables, and perceptual responses. Participants then consumed 500 mL of the assigned beverage and entered the environmental chamber for a 30 min passive heat exposure period to standardize pre-exercise environmental exposure before the time trial, consistent with prior heat-stress performance protocols incorporating controlled pre-time-trial chamber exposure [33]. Immediately before exercise, a second set of pre-exercise measurements was obtained.

Participants then completed a 5 min self-paced warm-up on the treadmill at 0% grade. After the warm-up, the treadmill was briefly stopped, and time and distance were reset before the 15 km time trial began. The treadmill gradient remained fixed at 0% throughout the trial. Participants were instructed to complete the 15 km distance as quickly as possible and were permitted to adjust treadmill speed at any time using the treadmill controls. Participants were made aware of the distance completed at 5 km intervals but were blinded to elapsed time, running speed, and overall performance time until both experimental trials were completed. Cumulative time was recorded at 5 km intervals and mean segment pace was calculated for the 0–5 km, 5–10 km, and 10–15 km segments.

During the time trial, respiratory gases, heart rate, estimated core temperature, and skin temperature were monitored continuously. Participants consumed 250 mL of the assigned beverage every 15 min of accumulated running time. During beverage administration and 5 km assessments, participants briefly straddled the treadmill while the assigned beverage was consumed, capillary blood glucose and lactate samples were collected, and perceptual ratings were obtained. Trial timing was paused during these brief interruptions and resumed when participants returned to running. Beverage administration required brief removal and replacement of the facemask, whereas the facemask remained in place during blood sampling and perceptual assessments unless these procedures coincided with a scheduled beverage dose. Interruptions were standardized between conditions and generally lasted ≤30 s. Immediately upon completion of the 15 km time trial, immediate post-exercise measurements were obtained, including capillary blood glucose and lactate, thermoregulatory variables, and perceptual responses. Participants then exited the chamber, towel-dried, changed into the same dry minimal clothing, and were reweighed. Additional post-exercise measurements, including urine specific gravity, blood pressure, capillary blood glucose and lactate, and perceptual responses, were obtained 10 min after time-trial completion.

### 2.11. Statistical Analysis

Statistical analyses were performed using SPSS version 28.0 (IBM Co., Armonk, NY, USA). Statistical significance was set a priori at *p* < 0.05. The primary outcome variable was the difference in final 15 km completion time between conditions and was analyzed using a paired-samples *t*-test. The mean paired difference, 95% confidence interval, and Cohen’s dz are reported. For *n* = 10, a sensitivity analysis for the paired primary outcome (α = 0.05, β = 0.20; two-tailed) indicated a minimum detectable effect of approximately Cohen’s dz = 1.00. Descriptive statistics were calculated for all variables and are reported as mean ± SD. Normality and absence of outliers were assessed using the Shapiro–Wilk test, normal Q-Q plots, and residual plots. All analyses were conducted using a two-tailed approach. Repeated-measures analyses of variance (RM ANOVA) were used to assess performance, physiological, metabolic, thermoregulatory, perceptual, cardiovascular, and hydration outcomes across conditions and time. Cumulative completion time was analyzed using a 2 × 3 RM ANOVA with condition (HSES vs. PLA) and distance (5, 10, and 15 km) as within-subject factors. Segment pace was analyzed using a 2 × 3 repeated-measures ANOVA with condition (HSES vs. PLA) and segment (0–5 km, 5–10 km, and 10–15 km) as within-subject factors. Segment-averaged physiological and metabolic variables during the time trial were analyzed using 2 × 3 RM ANOVA models. Capillary blood metabolites, thermoregulatory variables, and perceptual responses were analyzed using 2 × 6 RM ANOVA models across baseline, immediately before exercise, 5 km, 10 km, 15 km, and 10 min post-exercise. Body mass and urine specific gravity were analyzed using 2 × 2 RM ANOVA models, and blood pressure was analyzed using a 2 × 3 RM ANOVA. Training load and dietary intake variables were compared between trials using paired-samples *t*-tests. Bonferroni-adjusted post hoc comparisons were performed when significant main or interaction effects were observed. The assumption of sphericity was evaluated using Mauchly’s test, and Greenhouse–Geisser corrections were applied when this assumption was violated. Effect sizes were calculated using partial eta squared (ηp^2^) for ANOVA effects and Cohen’s *dz* for paired comparisons where appropriate. Cohen’s *dz* values were interpreted as trivial (<0.2), small (0.2–0.49), moderate (0.5–0.79), and large (≥0.8).

## 3. Results

### 3.1. Running Time-Trial Performance and Metabolic Responses

**Figure 1:**
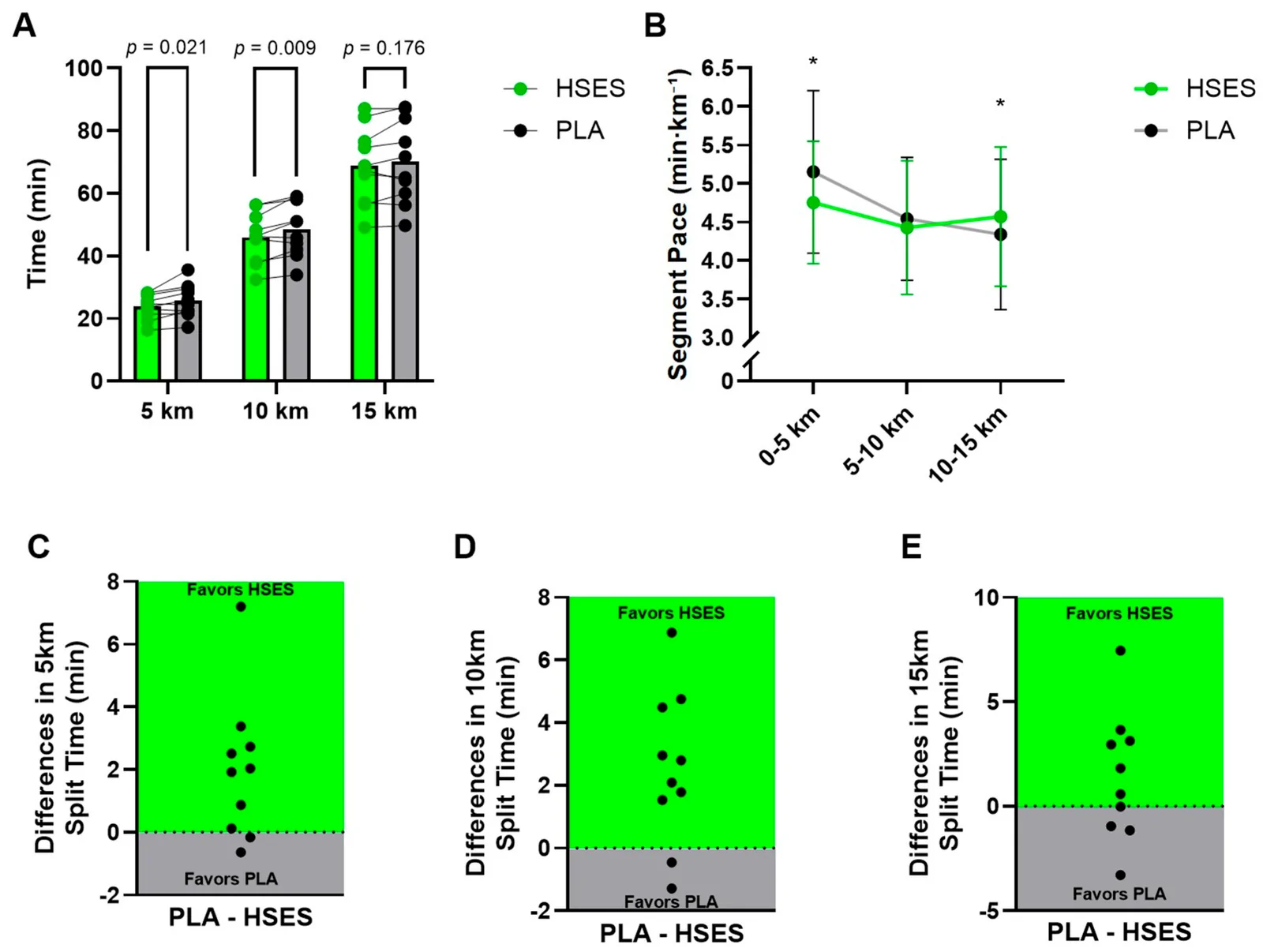
Fifteen-kilometer treadmill time-trial performance during high-sodium multi-electrolyte supplementation and placebo conditions. Panel (**A**) shows cumulative completion time at 5 km, 10 km, and 15 km during the high-sodium multi-electrolyte supplementation (HSES) and placebo (PLA) conditions. Bonferroni-adjusted post hoc comparisons following the repeated-measures analysis indicated faster cumulative times during HSES compared with PLA at 5 km (23.77 ± 3.97 vs. 25.77 ± 5.27 min; *p* = 0.021) and 10 km (45.92 ± 7.98 vs. 48.47 ± 8.69 min; *p* = 0.009), whereas the difference at 15 km (68.76 ± 12.28 vs. 70.18 ± 13.33 min; *p* = 0.176) was not statistically significant. These represented absolute differences of 2.00, 2.55, and 1.42 min and relative differences of 7.8%, 5.3%, and 2.0% at 5 km, 10 km, and 15 km, respectively. Panel (**B**) shows mean segment pace across the 0–5 km, 5–10 km, and 10–15 km segments. Lower values indicate faster pace. Segment pace differed between conditions during the 0–5 km and 10–15 km segments, with faster opening-segment pace during HSES and faster final-segment pace during PLA. Panels (**C**–**E**) show individual differences in cumulative time at 5 km, 10 km, and 15 km, respectively, calculated as PLA − HSES. Positive values indicate faster performance during HSES, whereas negative values indicate faster performance during PLA. At 5 km, 8 of 10 participants were faster during HSES; at 10 km, 8 of 10 were faster during HSES; and at 15 km, 6 of 10 were faster during HSES. Data are presented as mean ± SD with individual participant responses. * Symbols denote significant Bonferroni-adjusted between-condition comparisons: significantly different between HSES and PLA at the indicated segment (*p* < 0.05).

All participants (*n* = 10) were included in the final analysis. The results are summarized in Table 2 and Figure 1. Cumulative completion time showed a significant main effect of condition (*p* = 0.034, ηp^2^ = 0.410), a significant main effect of distance (*p* < 0.001, ηp^2^ = 0.996), and a significant condition × distance interaction (*p* = 0.023, ηp^2^ = 0.341). Bonferroni-adjusted follow-up comparisons indicated faster cumulative times during HSES than PLA at 5 km (23.77 ± 3.97 vs. 25.77 ± 5.27 min; *p* = 0.021) and 10 km (45.92 ± 7.98 vs. 48.47 ± 8.69 min; *p* = 0.009), whereas the difference at 15 km (68.76 ± 12.28 vs. 70.18 ± 13.33 min; *p* = 0.176) was not statistically significant. Because final 15 km completion time was the prespecified primary outcome, it was also analyzed separately using a paired-samples *t*-test. Mean final completion time was 68.76 ± 12.28 min during HSES and 70.18 ± 13.33 min during PLA. The mean paired difference (HSES − PLA) was −1.42 min (95% CI, −3.60 to 0.77 min), corresponding to an approximately 2.0% faster mean completion time during HSES; however, this difference was not statistically significant, t(9) = −1.47, *p* = 0.176, Cohen’s *dz* = −0.46 (95% CI, −1.11 to 0.20). Six of ten participants completed the 15 km time trial faster during HSES.

To further characterize pacing behavior, mean segment pace was calculated for each 5 km segment of the time trial (Figure 1B). There was no significant main effect of condition for segment pace (*p* = 0.180, ηp^2^ = 0.190), but there was a significant main effect of segment (*p* = 0.004, ηp^2^ = 0.453) and a significant condition × segment interaction (*p* = 0.001, ηp^2^ = 0.531). Mean segment pace during HSES was 4.75 ± 0.79, 4.43 ± 0.87, and 4.57 ± 0.90 min·km^−1^ for the 0–5 km, 5–10 km, and 10–15 km segments, respectively. Mean segment pace during PLA was 5.15 ± 1.05, 4.54 ± 0.80, and 4.34 ± 0.98 min·km^−1^, respectively. Follow-up comparisons indicated that HSES was associated with faster pace during the opening 0–5 km segment compared with PLA (*p* < 0.001), with no significant difference during the 5–10 km segment (*p* = 0.091), whereas PLA was associated with faster pace during the final 10–15 km segment compared with HSES (*p* = 0.032).

Segment-averaged physiological and metabolic responses are presented in Table 2. No significant condition effects or condition × time interactions were observed for HR, V̇O_2_, V̇CO_2_, V̇E, RER, carbohydrate oxidation, fat oxidation, or the percentage of total energy expenditure derived from carbohydrate or fat. Significant time effects were observed for HR (*p* = 0.043, ηp^2^ = 0.545) and V̇E (*p* = 0.020, ηp^2^ = 0.626). No other significant main effects or interactions were observed for the physiological or metabolic variables presented in Table 2.

### 3.2. Hydration Status and Sweat Rate

**Figure 2:**
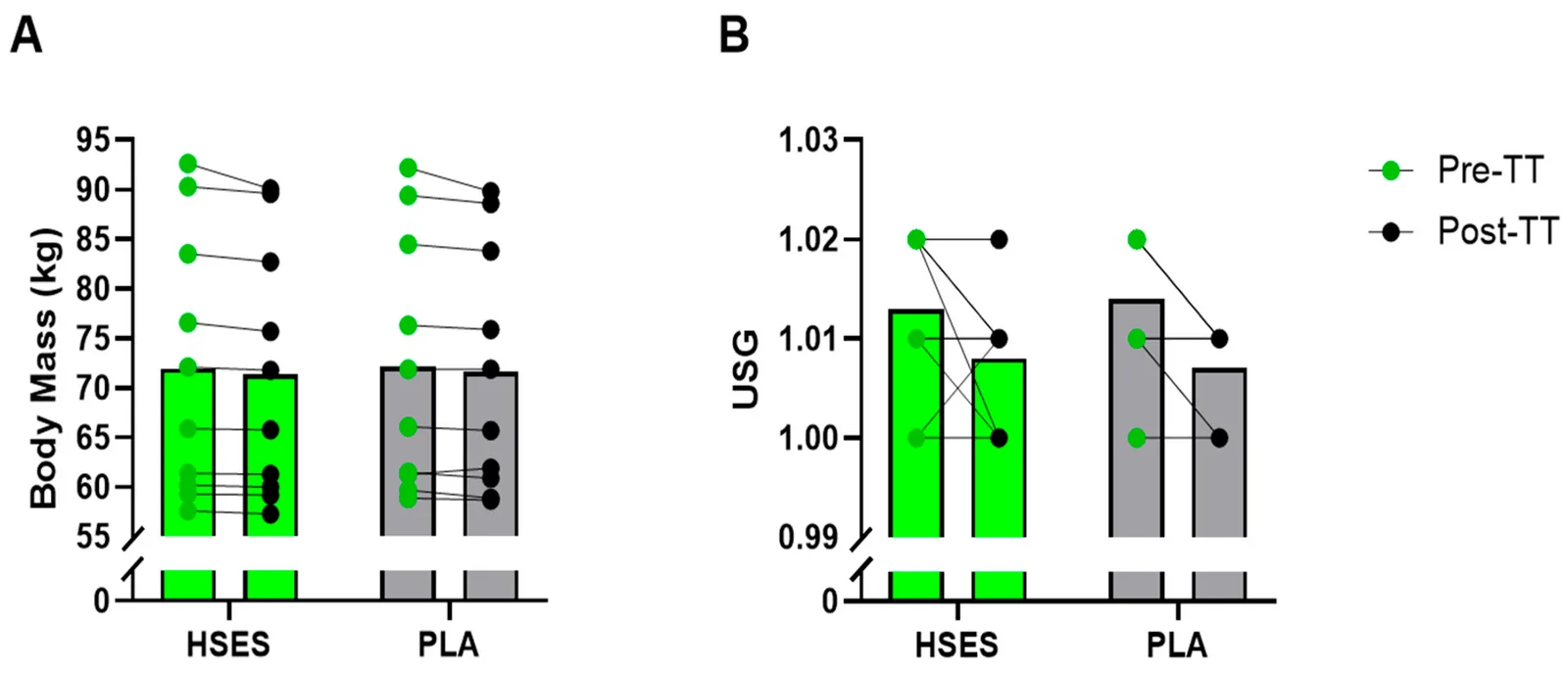
Body mass and urine specific gravity before and after the 15 km treadmill time trial. Panel (**A**) shows body mass before and after the time trial during the high-sodium multi-electrolyte supplementation (HSES) and placebo (PLA) conditions. Body mass decreased from pre- to post-time trial in both conditions (HSES: 71.9 ± 13.2 to 71.4 ± 12.6 kg; PLA: 72.2 ± 12.8 to 71.6 ± 12.3 kg), with a significant main effect of time (*p* = 0.031), but no condition effect (*p* = 0.302) or condition × time interaction (*p* = 0.799). Panel (**B**) shows urine specific gravity (USG) before and after the time trial during HSES and PLA. USG decreased from pre- to post-time trial in both conditions (HSES: 1.013 ± 0.007 to 1.008 ± 0.004; PLA: 1.014 ± 0.007 to 1.007 ± 0.004), with a significant main effect of time (*p* < 0.001), but no condition effect (*p* = 0.866) or condition × time interaction (*p* = 0.809). Data are presented as mean ± SD with individual participant responses.

Body mass and USG responses are presented in Figure 2 and Table S3. Participants arrived for both trials in a similar hydration state, with pre-time-trial USG values below 1.020 during both HSES and PLA. Body mass decreased from pre- to post-time trial across conditions, as indicated by a significant main effect of time (*p* = 0.031, ηp^2^ = 0.419), with no significant condition effect (*p* = 0.302, ηp^2^ = 0.117) or condition × time interaction (*p* = 0.799, ηp^2^ = 0.008). Specifically, body mass decreased from 71.9 ± 13.2 to 71.4 ± 12.6 kg during HSES and from 72.2 ± 12.8 to 71.6 ± 12.3 kg during PLA. Expressed relative to pre-time-trial body mass, this corresponded to body mass losses of approximately 0.85% during HSES and 0.79% during PLA.

USG also decreased from pre- to post-time trial across conditions, with a significant main effect of time (*p* < 0.001, ηp^2^ = 0.854), but no significant condition effect (*p* = 0.866, ηp^2^ = 0.003) or condition × time interaction (*p* = 0.809, ηp^2^ = 0.007). USG decreased from 1.013 ± 0.007 to 1.008 ± 0.004 during HSES and from 1.014 ± 0.007 to 1.007 ± 0.004 during PLA. Mean total fluid intake was 1.50 ± 0.17 L during HSES and 1.60 ± 0.17 L during PLA. Sweat rate did not differ between conditions (HSES: 1.64 ± 0.44 L·h^−1^; PLA: 1.66 ± 0.58 L·h^−1^; *p* = 0.871, d = −0.04).

### 3.3. Thermoregulatory Responses

**Figure 3:**
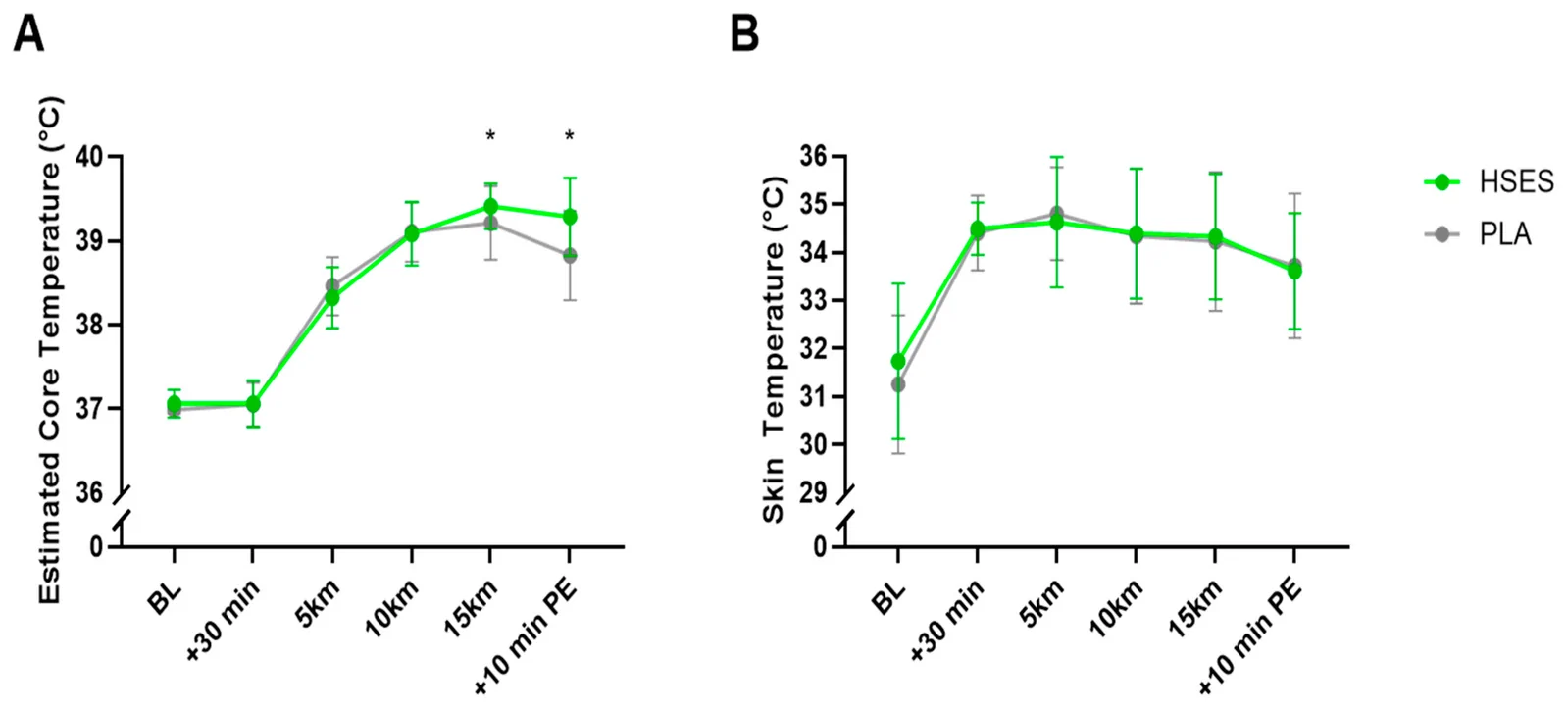
Thermoregulatory responses during the 15 km treadmill time trial. Panel (**A**) shows estimated core temperature (Tc) and Panel (**B**) shows skin temperature (Tsk) during high-sodium multi-electrolyte supplementation (HSES) and placebo (PLA) conditions. Estimated Tc increased over time in both conditions and was significantly higher during HSES compared with PLA at 15 km (39.4 ± 0.3 vs. 39.2 ± 0.4 °C) and 10 min post-exercise (39.3 ± 0.4 vs. 38.8 ± 0.6 °C). Tsk increased from baseline to exercise and remained elevated throughout the time trial, with no significant condition effect or condition × time interaction. Data are presented as mean ± SD. BL = baseline; +30 min = immediately before exercise; 5 km = 5 kilometers; 10 km = 10 kilometers; 15 km = 15 kilometers; +10 min PE = 10 minutes post-exercise. * = *p* < 0.05 between conditions at the indicated timepoint.

Estimated core temperature (Tc) and skin temperature (Tsk) responses are presented in Figure 3 and Table S4. Estimated Tc increased over time during the 15 km time trial in both conditions, with significant main effects for time (*p* < 0.001, ηp^2^ = 0.968) and condition (*p* = 0.033, ηp^2^ = 0.413), as well as a significant condition × time interaction (*p* < 0.001, ηp^2^ = 0.414). Estimated Tc was similar between conditions at baseline, immediately before exercise, at 5 km, and at 10 km. Post hoc comparisons indicated that estimated Tc was significantly higher during HSES compared with PLA at 15 km (39.4 ± 0.3 vs. 39.2 ± 0.4 °C; *p* = 0.022) and 10 min post-exercise (39.3 ± 0.4 vs. 38.8 ± 0.6 °C; *p* < 0.001).

Tsk also changed over time, with a significant main effect of time (*p* < 0.001, ηp^2^ = 0.756). Tsk increased from baseline to immediately before exercise and remained elevated throughout the time trial. No significant condition effect (*p* = 0.766, ηp^2^ = 0.010) or condition × time interaction (*p* = 0.506, ηp^2^ = 0.053) was observed for Tsk.

### 3.4. Blood Pressure Responses

Systolic and diastolic blood pressure responses are presented in Table S5. Systolic blood pressure decreased over time, with a significant main effect of time (*p* = 0.002, ηp^2^ = 0.512), but no significant condition effect (*p* = 0.378, ηp^2^ = 0.087) or condition × time interaction (*p* = 0.947, ηp^2^ = 0.006). Diastolic blood pressure also decreased over time, with a significant main effect of time (*p* = 0.008, ηp^2^ = 0.413), but no significant condition effect (*p* = 0.960, ηp^2^ = 0.000) or condition × time interaction (*p* = 0.847, ηp^2^ = 0.018).

### 3.5. Capillary Blood Metabolite Responses

The results for capillary blood glucose and lactate concentrations are summarized in Table 3. Blood glucose concentrations increased over time during the 15 km time trial, with a significant main effect of time (*p* < 0.001, ηp^2^ = 0.434). However, no significant main effect of condition (*p* = 0.155, ηp^2^ = 0.212) or condition × time interaction (*p* = 0.749, ηp^2^ = 0.056) was observed. Glucose concentrations increased from baseline to 15 km in both conditions, rising from 97.6 ± 7.8 to 135.5 ± 54.3 mg·dL^−1^ during HSES and from 96.1 ± 8.4 to 129.2 ± 35.4 mg·dL^−1^ during PLA.

Capillary blood lactate concentrations also increased over time, with a significant main effect of time (*p* < 0.001, ηp^2^ = 0.624). There was no significant main effect of condition (*p* = 0.076, ηp^2^ = 0.308) or condition × time interaction (*p* = 0.332, ηp^2^ = 0.116). Lactate concentrations increased from baseline to 15 km in both conditions, rising from 1.3 ± 0.4 to 3.3 ± 1.5 mmol·L^−1^ during HSES and from 1.1 ± 0.3 to 3.3 ± 0.7 mmol·L^−1^ during PLA.

### 3.6. Perceptual Responses

Perceptual responses are summarized in Table 4. RPE, affect, thermal sensation, and GI distress changed significantly over time during the 15 km time trial (all time effects, *p* < 0.001). RPE increased progressively during exercise, reaching 8.5 ± 1.0 during HSES and 8.5 ± 0.7 during PLA at 15 km. Affect became more negative across the trial, reaching −2.5 ± 1.7 during HSES and −2.9 ± 1.4 during PLA at 15 km. Thermal sensation also increased similarly between conditions, reaching 3.3 ± 0.7 during HSES and 3.3 ± 0.9 during PLA at 15 km, while GI distress increased to 4.2 ± 2.4 and 4.3 ± 3.1 during HSES and PLA, respectively.

No significant condition effects were observed for RPE, affect, thermal sensation, or GI distress (all *p* ≥ 0.191), and no condition × time interactions were observed for these variables (all *p* ≥ 0.314). Thirst did not significantly change over time (*p* = 0.051), did not differ between conditions (*p* = 0.073), and showed no condition × time interaction (*p* = 0.691). At 15 km, thirst ratings were 4.0 ± 1.5 during HSES and 3.9 ± 1.5 during PLA.

## 4. Discussion

### 4.1. Main Findings

There were two key findings of this study. First, cumulative completion times were significantly faster during HSES at 5 and 10 km, whereas the difference at the final 15 km endpoint was not statistically significant. Segment pace also differed across the trial, with a faster opening 0–5 km during HSES and a faster final 10–15 km during PLA. Mean final completion time was 1.42 min (~2.0%) faster during HSES, although this difference was not statistically significant. Second, no significant between-condition differences were detected in the measured hydration-related, cardiovascular, metabolic, or perceptual outcomes. Estimated core temperature was higher during HSES late in exercise and during early recovery.

The study was designed to evaluate whether a carbohydrate-free, high-sodium multi-electrolyte beverage could influence running time-trial performance under warm-humid conditions independent of exogenous carbohydrate provision. Previous beverage studies have examined performance responses to fluid and carbohydrate provision, both separately and in combination [34,35,36]. In contrast, the present study used a carbohydrate-free HSES beverage and a flavor-matched, electrolyte-free PLA beverage, thereby removing exogenous carbohydrate as a between-condition difference. Many sodium and hyperhydration studies have also used large-volume pre-exercise hyperhydration, cycling protocols, or fixed-intensity exercise-to-exhaustion models; the present design therefore provided a practical test of repeated HSES ingestion during self-paced running in a warm-humid environment [17,31,37].

### 4.2. Time-Trial Performance and Pacing

The first important finding was that cumulative completion times were significantly faster during HSES at 5 and 10 km, but not at the final 15 km endpoint. The repeated-measures analysis showed a significant condition effect and condition × distance interaction. The mean separation between conditions was 2.00 min at 5 km, increased to 2.55 min at 10 km, and narrowed to 1.42 min at 15 km. The segment-pace analysis further showed that participants ran faster during HSES over the opening 0–5 km, there was no significant difference during 5–10 km, and participants ran faster during PLA over the final 10–15 km. Thus, much of the cumulative separation was established early in the trial and subsequently narrowed toward the finish. However, given the small sample and limited precision of the effect estimate, the nonsignificant final 15 km comparison should not be interpreted as evidence of no effect.

The present findings should be considered alongside previous investigations of sodium loading and sodium-induced hyperhydration in the heat, which have produced variable performance responses across exercise models. Sims et al. [38] reported that pre-exercise sodium loading increased plasma volume and improved running time to exhaustion in trained men exercising in the heat, while Morris et al. [39] found that acute sodium ingestion before exercise increased voluntary water consumption, improved fluid retention, and improved cycling time-trial performance in warm conditions. Similarly, Hamouti et al. [15] observed that sodium plus water ingestion before prolonged cycling better maintained plasma volume and cardiovascular function and increased mean work rate during the subsequent cycling time trial, whereas Convit et al. [16] reported that sodium hyperhydration improved cycling time-trial performance in hot-humid conditions despite no clear differences in thermal or cardiovascular strain. In contrast, Gigou et al. [17] reported that sodium-induced hyperhydration increased body mass and reduced physiological strain but did not improve 18 km treadmill running time-trial performance under warm conditions, while Dion et al. [31] showed that programmed fluid intake reduced body mass loss, rectal temperature, and heart rate during a half-marathon treadmill time trial but did not improve performance compared with thirst-driven intake.

Collectively, these studies indicate that the ergogenic potential of sodium or hyperhydration strategies depends heavily on the exercise model, fluid volume, environmental conditions, and performance outcome. In their systematic review, Jardine et al. [37] concluded that pre-exercise hyperhydration appears more consistently beneficial for exercise capacity during constant work-rate exercise than for time-trial performance, where pacing strategy, exercise modality, and changes in body mass may influence the outcome. However, a more recent meta-analysis by McCubbin and Irwin [40], which included only studies that demonstrated a measurable difference in pre-exercise hydration status between conditions, reported small beneficial effects of hyperhydration on both TTE and TT performance. This distinction is important because the performance effect of sodium or hyperhydration strategies may depend not only on the osmotic agent used but also on whether the intervention produces a meaningful difference in fluid retention before exercise. This is especially relevant for running, where a large increase in retained fluid could theoretically increase the energetic cost of weight-bearing exercise and offset some of the cardiovascular or thermoregulatory benefit of hyperhydration. The present study did not use a large-volume pre-exercise hyperhydration protocol and did not show evidence of greater body mass change between conditions. The present protocol therefore differed from traditional hyperhydration approaches by using a 500 mL pre-exercise dose followed by repeated in-trial ingestion, rather than a large-volume pre-exercise fluid load, and by providing no exogenous carbohydrate.

### 4.3. Mechanistic Considerations

The mechanisms underlying the observed differences in cumulative performance and pacing are not clear from the measurements included in this study. It is usually proposed that sodium ingestion during exercise influences endurance performance through effects on fluid retention, plasma volume, cardiovascular strain, thermoregulation, and perceptual responses [15,37,38,40]. However, no significant between-condition differences were observed in body mass change, USG, sweat rate, blood pressure, heart rate, respiratory gas exchange, substrate oxidation, blood glucose, blood lactate concentrations, RPE, affect, thermal sensation, thirst, or GI distress. Thus, the observed performance and pacing differences were not accompanied by differences in the traditional whole-body hydration, cardiovascular, metabolic, or perceptual outcomes measured in this study. Furthermore, because HSES contained sodium, potassium, and magnesium, the contribution of any individual electrolyte cannot be determined.

One possible explanation is that HSES produced subtle changes in fluid distribution or intravascular fluid support that were not captured by body mass, USG, or sweat rate. Sodium is the primary extracellular cation and plays an important role in plasma osmolality, renal water retention, thirst regulation, and extracellular fluid volume [10,11]. In sodium and hyperhydration studies, improved performance or exercise capacity has often been attributed to greater fluid retention, expanded plasma volume, and improved maintenance of cardiovascular function during exercise in the heat [15,37,38,39,40]. However, sodium balance during prolonged exercise is complex and depends on the interaction between fluid intake, sweat sodium losses, sodium ingestion, and changes in body water compartments [8,11,41]. Therefore, the lack of differences in body mass change, USG, or sweat rate does not exclude differences in extracellular fluid distribution or plasma volume that were not captured by these measures. The prescribed in-trial beverage schedule provided approximately 1.0 L·h^−1^, which was lower than the measured sweat rate in both conditions. Thus, the protocol did not replace all sweat losses, and the small difference in total fluid intake between conditions was not accompanied by differences in body mass change, USG, or sweat rate. Nevertheless, because plasma volume, serum sodium concentrations, plasma osmolality, and sweat sodium concentration were not measured, any explanation involving altered intravascular volume or sodium–water balance remains speculative.

Heart rate increased across the time trial and blood pressure changed over time, but neither HR nor BP differed between HSES and PLA. This differs from some sodium-loading studies in which improved exercise capacity was accompanied by lower heart rate or reduced cardiovascular strain at a fixed workload [37,38]. However, the present study used a self-paced time trial rather than fixed-intensity exercise. In this context, similar HR values between conditions do not necessarily indicate identical exercise stress because running speed was self-selected and differed across segments. McCubbin and Irwin [40] similarly reported no clear overall effect of pre-exercise hyperhydration on HR at the conclusion of performance tasks and noted that, when an intervention improves physiological capacity, participants may perform more work or sustain a faster pace, thereby offsetting reductions in HR that might otherwise be observed during fixed-intensity exercise. Thus, the HR and BP responses measured in the present study do not identify a cardiovascular mechanism for the observed pacing differences. This interpretation is similar to that proposed by Convit et al. [16], who reported improved cycling time-trial performance following sodium hyperhydration despite no clear differences in heart rate or rectal temperature.

The carbohydrate-free nature of HSES is also important when interpreting the metabolic findings. Capillary glucose and lactate concentrations increased over time during the time trial but did not differ between conditions. Likewise, V̇O_2_, V̇CO_2_, V̇E, RER, and calculated carbohydrate and fat oxidation did not differ between HSES and PLA. Thus, the observed between-condition differences in pacing and cumulative time occurred in the absence of exogenous carbohydrate provision and were not accompanied by detectable differences in blood glucose concentration or whole-body substrate oxidation.

An additional hypothesis is that HSES influenced performance through oral and/or post-ingestive sensory signaling with downstream effects on central motor regulation. This interpretation is consistent with mouth rinse studies showing that carbohydrate can influence exercise performance, brain activity, and motor output before it contributes meaningfully as an oxidizable fuel, and that this effect may not be unique to carbohydrate [42,43]. Khong et al. [44] reported that both glucose and NaCl mouth rinses attenuated reductions in maximal voluntary contraction and sustained maximal voluntary contraction following fatiguing cycling exercise compared with water. Because the solutions were expectorated, these responses occurred without ingestion, supporting the possibility of an oral-sensory contribution. More recently, Han et al. [45] showed that carbohydrate and salt mouth rinses better preserved maximal voluntary contraction, sustained maximal voluntary contraction, voluntary activation, electromyography, and central activation ratio compared with placebo, consistent with an attenuation of central fatigue. The present study involved ingestion rather than mouth rinsing and did not directly assess voluntary activation, corticospinal excitability, electromyography, motor unit recruitment, or muscle mechanical properties. Therefore, oral sensory signaling remains a hypothesis-generating explanation for the early pacing difference and cannot be established from the present measurements.

Expectancy could also have influenced initial pace selection. An intervention designed to increase expectancy of ergogenicity improved exercise capacity without corresponding differences in heart rate or blood metabolites [46]. Because treatment identification was not formally quantified and complete taste matching was not possible, the present study cannot distinguish expectancy from oral-sensory or post-ingestive effects.

### 4.4. Thermoregulatory and Perceptual Responses

Thermoregulatory responses should be interpreted cautiously. Estimated core temperature measured with the CORE sensor was higher with HSES late in the time trial and during early recovery, whereas skin temperature and thermal sensation did not differ between conditions. In this self-paced protocol, the higher estimated Tc late in HSES may partly reflect greater metabolic heat production associated with the faster pace selected earlier in the trial rather than a direct thermoregulatory effect of HSES. However, the CORE sensor provides a noninvasive estimate of core temperature, and validation studies have reported mixed agreement with criterion measures during exercise in the heat [30,47,48,49]. Therefore, the higher estimated core temperature observed during HSES should not be interpreted as definitive evidence of greater thermoregulatory strain. Future studies should confirm these responses using rectal, esophageal, or ingestible telemetry methods.

Perceptual responses did not differ significantly between conditions despite the differences in pacing. RPE, affect, thermal sensation, and GI distress changed over time during prolonged running under warm-humid conditions, but no condition × time interactions were observed. Thirst did not change significantly over time and did not differ between conditions. The lack of a between-condition difference in RPE is notable because participants selected a faster opening pace during HSES while reporting similar perceived exertion. However, similar RPE does not establish that HSES physiologically enabled a higher running speed. This is consistent with the findings of McCubbin and Irwin [40], who reported no clear overall effect of pre-exercise hyperhydration on RPE despite evidence of improved endurance performance. The absence of a between-condition difference in GI distress is also relevant because hyperhydration and high-sodium strategies can increase gastrointestinal symptoms depending on dose, timing, fluid volume, and ingestion rate [37]. In the present study, participants received HSES before and repeatedly during the time trial, resulting in a mean total intake of approximately 3000 mg sodium, 600 mg potassium, and 180 mg magnesium, yet GI distress did not differ significantly from PLA. Thus, despite the relatively high cumulative electrolyte intake during HSES, the GI distress response was similar between conditions.

### 4.5. Strengths and Limitations

The present study has several strengths, including the randomized counterbalanced crossover design, placebo control, pretrial dietary and training replication procedures, and controlled warm-humid testing conditions. Several limitations should also be acknowledged. The small sample limits the precision of the primary performance estimate and the ability to detect effects of the magnitude observed in the present study; it also limits statistical sensitivity for secondary outcomes. Complete taste matching was not possible, and treatment identification was not formally quantified; therefore, expectancy effects cannot be excluded. The multi-electrolyte formulation does not permit attribution to an individual electrolyte. Total fluid intake was slightly greater during PLA as a consequence of the time-based dosing schedule. Plasma volume, serum sodium, plasma osmolality, sweat sodium, and neuromuscular activation were not measured, limiting mechanistic interpretation, and core temperature was estimated rather than measured using a criterion method. Dietary intake was self-reported, although no statistically significant between-trial differences were detected. Finally, the sample comprised trained male runners, and the treadmill trials included brief measurement interruptions, limiting generalization to other populations and uninterrupted competition.

### 4.6. Conclusions

In trained male runners exercising at 26.7 °C and 60% RH, pacing differed between conditions during the 15 km treadmill time trial, with a faster opening 5 km during HSES and a faster final 5 km during PLA. Cumulative completion time was significantly faster during HSES at 5 and 10 km, whereas final 15 km completion time was approximately 2.0% faster but did not differ significantly between conditions. Given the small sample and limited precision of the effect estimate, this nonsignificant finding should not be interpreted as evidence of no effect. No significant between-condition differences were detected in the measured hydration-related, cardiovascular, metabolic, or perceptual outcomes. Estimated core temperature was higher during HSES late in exercise and early recovery. The mechanisms underlying the observed pacing and performance responses, including any contribution from sensory or expectancy effects, remain unresolved.

## Figures and Tables

**Table 1:** Participant demographics (*n* = 10).

Characteristics	Mean ± SD
Age (years)	29.6 ± 11.9
Height (cm)	173.9 ± 5.7
Body Mass (kg)	72.1 ± 12.7
Body Fat (%)	9.6 ± 4.3
BMI (kg/m^2^)	23.6 ± 3.2
Fat Mass (kg)	9.7 ± 9.4
Fat Free Mass (kg)	64.2 ± 8.7
Running Experience (years)	9.3 ± 5.9
Weekly Distance (km)	66.1 ± 29.1
VO_2_max (mL/kg/min)	57.3 ± 8.1

Data shown as mean ± SD. BMI, Body Mass Index; VO_2_max; Maximal Oxygen Consumption.

**Table 2:** Physiological, metabolic, respiratory, and performance data collected during the time trial (*n* = 10).

Variable	Condition	Timepoint			*2 × 3* RM ANOVA *p*-Value; ηp^2^		
		5 km	10 km	15 km	Condition	Time	Interaction
**Cumulative Time (min)**	**HSES**	23.77 ± 3.97 *	45.92 ± 7.98 *	**68.76 ± 12.28**	**0.034** **; 0.410**	**<0.001** **; 0.996**	**0.023** **; 0.341**
	**PLA**	**25.77 ± 5.27**	**48.47 ± 8.69**	**70.18 ± 13.33**			
**HR (b·min^−1^)**	**HSES**	**166.5 ± 20.0**	**176.1 ± 11.4**	**178.9 ± 9.3**	**0.823; 0.006**	**0.043** **; 0.545**	**0.195; 0.335**
	**PLA**	**163.9 ± 18.1**	**177.4 ± 12.3**	**180.7 ± 11.24**			
**V̇O_2_** **(L·min^−1^)**	**HSES**	**3.13 ± 0.36**	**3.07 ± 0.32**	**3.32 ± 0.35**	**0.182; 0.189**	**0.120; 0.412**	**0.308; 0.255**
	**PLA**	**2.96 ± 0.52**	**3.09 ± 0.37**	**3.15 ± 0.34**			
**V̇O_2_** **(mL·kg^−1^·min^−1^)**	**HSES**	**44.81 ± 9.69**	**43.80 ± 8.64**	**47.31 ± 9.05**	**0.111; 0.258**	**0.179; 0.350**	**0.398; 0.206**
	**PLA**	**41.97 ± 6.59**	**43.48 ± 6.86**	**44.29 ± 6.34**			
**V̇CO_2_** **(L·min^−1^)**	**HSES**	**2.80 ± 0.39**	**2.70 ± 0.28**	**3.01 ± 0.36**	**0.401; 0.080**	**0.124; 0.406**	**0.315; 0.251**
	**PLA**	**2.65 ± 0.48**	**2.77 ± 0.38**	**2.85 ± 0.33**			
**V̇E** **(L·min^−1^)**	**HSES**	**97.2 ± 14.52**	**97.93 ± 9.91**	**110.42 ± 13.0**	**0.373; 0.089**	**0.020** **; 0.626**	**0.175; 0.353**
	**PLA**	**82.26 ± 29.37**	**102.65 ± 16.08**	**108.02 ± 13.49**			
**RER**	**HSES**	**0.89 ± 0.04**	**0.88 ± 0.02**	**0.91 ± 0.04**	**0.520; 0.047**	**0.160; 0.367**	**0.559; 0.136**
	**PLA**	**0.90 ± 0.03**	**0.90 ± 0.03**	**0.91 ± 0.04**			
**CHO oxidation (g·min^−1^)**	**HSES**	**2.67 ±0.71**	**2.44 ± 0.33**	**3.04 ± 0.71**	**0.947; 0.001**	**0.126; 0.405**	**0.330; 0.242**
	**PLA**	**2.55 ± 0.62**	**2.68 ± 0.66**	**2.89 ± 0.67**			
**FAT oxidation (g·min^−1^)**	**HSES**	**0.56 ± 0.15**	**0.61 ± 0.13**	**0.52 ± 0.22**	**0.302; 0.118**	**0.154; 0.374**	**0.580; 0.127**
	**PLA**	**0.52 ± 0.14**	**0.53 ± 0.17**	**0.49 ± 0.22**			
**%Total EE CHO**	**HSES**	**64.0 ± 12.65**	**60.33 ± 6.75**	**68.33 ± 13.26**	**0.520; 0.047**	**0.160; 0.367**	**0.559; 0.135**
	**PLA**	**65.0 ± 10.21**	**65.33 ± 11.57**	**69.67 ± 14.27**			
**%Total EE Fat**	**HSES**	**36.0 ± 12.65**	**39.67 ± 6.75**	**31.67 ± 13.26**	**0.520; 0.047**	**0.160; 0.367**	**0.559; 0.135**
	**PLA**	**35.0 ± 10.21**	**34.67 ±11.57**	**30.34 ±14.27**			

Data are shown as mean ± SD (*n* = 10). Cumulative completion time is presented at 5 km, 10 km, and 15 km. Physiological and metabolic variables were averaged across each 5 km segment of the 15 km treadmill time trial. HR = heart rate; V̇O_2_ = oxygen consumption; V̇CO_2_ = carbon dioxide production; V̇E = ventilation; RER = respiratory exchange ratio; CHO = carbohydrate; EE = energy expenditure; HSES = high-sodium multi-electrolyte supplementation; PLA = placebo; ηp^2^ = partial eta squared. Bolded *p* values indicate statistically significant main or interaction effects. * = *p* < 0.05 between conditions at the indicated distance.

**Table 3:** Capillary blood metabolite responses.

Variable	Condition	Timepoint						2 × 6 RM ANOVA *p*-Value; ηp^2^
		BL	IBE	5 km	10 km	15 km	10PE	
Glucose (mg·dL^−1^)	HSES	97.6 ± 7.8	96.6 ± 7.5	122.1 ± 24.2	133.7 ± 37.7	135.5 ± 54.3	114.7 ± 37.1	Time, ***p* < 0.001;** 0.434 Condition, *p* = 0.155; 0.212 Interaction, *p* = 0.749; 0.056
	PLA	96.1 ± 8.4	96.7 ± 9.1	118.5 ± 30.9	131.4 ± 36.6	129.2 ± 35.4	103.0 ± 23.1	
Lactate (mmol·L^−1^)	HSES	1.3 ± 0.4	1.5 ± 0.4	3.3 ± 1.4	2.6 ± 1.1	3.3 ± 1.5	2.1 ± 0.7	Time, ***p* < 0.001;** 0.624 Condition, *p* = 0.076; 0.308 Interaction, *p* = 0.332; 0.116
	PLA	1.1 ± 0.3	1.4 ± 0.6	2.4 ± 1.1	2.6 ± 0.7	3.3 ± 0.7	1.8 ± 0.6	

Data are shown as mean ± SD (*n* = 10). Finger capillary blood glucose and lactate were assessed across six timepoints (*n* = 10). Glucose is reported in mg·dL^−1^, and lactate is reported in mmol·L^−1^. BL = baseline; IBE = immediately before exercise; 5 km = 5 kilometers; 10 km = 10 kilometers; 15 km = 15 kilometers; 10PE = 10 minutes post-exercise; HSES = high-sodium multi-electrolyte supplementation; PLA = placebo; ηp^2^ = partial eta squared. Boldface values denote a significant effect (*p* < 0.05).

**Table 4:** Perceptual responses.

Variable	Condition	Timepoint						2 × 6 RM ANOVA *p*-Value; ηp^2^
		BL	IBE	5 km	10 km	15 km	10PE	
RPE	HSES	0.2 ± 0.4	0.3 ± 0.7	6.2 ± 1.1	7.6 ± 1.1	8.5 ± 1.0	0.7 ± 1.1	Time, ***p* < 0.001;** 0.964 Condition, *p* = 0.191; 0.182 Interaction, *p* = 0.314; 0.120
	PLA	0.4 ± 0.7	0.3 ± 0.7	6.4 ± 0.7	7.7 ± 0.7	8.5 ± 0.7	1.5 ± 1.9	
Affect	HSES	1.8 ± 1.5	1.9 ± 1.7	0.0 ± 1.3	−1.6 ± 1.2	−2.5 ± 1.7	1.6 ± 1.8	Time, ***p* < 0.001;** 0.774 Condition, *p* = 0.474; 0.058 Interaction, *p* = 0.682; 0.065
	PLA	2.5 ± 1.4	2.2 ± 1.4	0.5 ± 1.4	−1.4 ± 2.1	−2.9 ± 1.4	1.7 ± 1.6	
Thermal Sensation	HSES	0.1 ± 0.9	0.5 ± 1.0	2.2 ± 0.6	3.0 ± 0.7	3.3 ± 0.7	0.7 ± 1.3	Time, ***p* < 0.001;** 0.848 Condition, *p* = 0.744; 0.014 Interaction, *p* = 0.953; 0.027
	PLA	−0.2 ± 0.8	0.6 ± 0.8	2.2 ± 0.4	2.9 ± 0.3	3.3 ± 0.9	0.7 ± 1.3	
Thirst	HSES	3.7 ± 1.3	3.5 ± 1.0	4.2 ± 1.1	4.3 ± 1.6	4.0 ± 1.5	4.2 ± 1.1	Time, *p* = 0.051; 0.211 Condition, *p* = 0.073; 0.314 Interaction, *p* = 0.691; 0.064
	PLA	3.5 ± 1.5	2.9 ± 1.3	3.6 ± 1.3	2.8 ± 1.8	3.9 ± 1.5	3.7 ± 1.4	
GI Distress	HSES	0.4 ± 0.5	0.6 ± 0.7	1.7 ± 1.6	2.6 ± 2.1	4.2 ± 2.4	1.2 ± 1.2	Time, ***p* < 0.001;** 0.624 Condition, *p* = 0.310; 0.128 Interaction, *p* = 0.359; 0.124
	PLA	0.0 ± 0.0	0.7 ± 1.1	2.0 ± 1.9	3.2 ± 2.3	4.3 ± 3.1	1.4 ± 2.1	

Data are shown as mean ± SD (*n* = 10). Perceptual responses were assessed across six timepoints (*n* = 10). RPE was assessed using the OMNI Walk/Run scale, ranging from 0 “extremely easy” to 10 “extremely hard.” Affect was assessed using the Feeling Scale, ranging from −5 “very bad” to +5 “very good.” Thermal sensation was assessed using a 9-point scale, ranging from −4 “very cold” to +4 “very hot.” Thirst was assessed using a 7-point scale, ranging from 1 “not thirsty at all” to 7 “very, very thirsty.” GI distress was assessed using a 0–10 scale, ranging from 0 “none” to 10 “severe.” BL = baseline; IBE = immediately before exercise; 5 km = 5 kilometers; 10 km = 10 kilometers; 15 km = 15 kilometers; 10PE = 10 minutes post-exercise; HSES = high-sodium multi-electrolyte supplementation; PLA = placebo; ηp^2^ = partial eta squared. Boldface values denote a significant effect (*p* < 0.05).

## Data Availability

The raw data supporting the conclusions of this article will be made available by the authors on request.

## Supplementary Materials

The following supporting information can be downloaded at https://www.mdpi.com/article/10.3390/sports14100435/s1: Table S1: Training load; Table S2: Dietary intake log; Table S3: Body mass and hydration status; Table S4: Thermoregulatory responses; Table S5: Blood pressure responses.
