# Supplementary material for: Effects of a High-Sodium, Carbohydrate-Free Multi-Electrolyte Supplement on 15 km Treadmill Time-Trial Performance in Warm and Humid Conditions: A Randomized, Single-Blind, Placebo-Controlled Crossover Trial
Source: Sports (Basel). 2026 Oct 7;14(10):435. doi: 10.3390/sports14100435 (PMC13709417; doi:10.3390/sports14100435)
Supplement: Supplementary file 1 [file sports-14-00435-s001.zip › sports-4547522-supplementary.pdf]

Supplementary Materials

Supplementary Tables

Table S1 | Training Load

Variable	Trial 1	Trial 2	*p*-value
**Training Load (RPE x min)**	2068 ± 1355	2020 ± 1226	0.355

Data shown as mean ± SD. Participants (*n* = 10) recorded their training load using the session-RPE method (RPE × session duration in minutes) during the weeks leading to experimental sessions. No statistically significant difference in training load was detected between experimental trials.

Table S2. Dietary Intake Log

Variable	Trial 1	Trial 2	*p*-value
Total Energy Consumed (kcal)	2676 ± 590	2528 ± 606	0.322
Carbohydrate (g)	315 ± 130	262 ± 59	0.179
Protein (g)	158 ± 89	146 ± 70	0.659
Fat (g)	114 ± 40	105 ± 33	0.484
Carbohydrate (%)	47 ± 14	43 ± 9	0.378
Protein (%)	23 ± 10	23 ± 8	0.912
Fat (%)	38 ± 7	37 ± 6	0.824
Cholesterol (mg)	640 ± 494	537 ± 454	0.344
Fiber (g)	19 ±11	18 ± 4	0.703

Sugar (g)	85 ±37	77 ± 35	0.721

Participants (*n* = 10) recorded dietary intake during the three days preceding each experimental trial using MyFitnessPal. Participants were instructed to maintain their habitual dietary pattern and to replicate dietary intake as closely as possible before each experimental trial. Data reflect average daily intake. No significant differences were observed between trials. Values are presented as mean ± SD.

Table S3 | Body Mass and Hydration Status

Variable	HSES		PLA		2 × 2 RM ANOVA *P-Value*; η2p		
	Pre TT	Post TT	Pre TT	Post TT	Condition	Time	Interaction
**Body mass (kg)**	71.9 ± 13.2	71.4 ± 12.6	72.2 ± 12.8	71.6 ± 12.3	0.302; 0.117	**0.031**; 0.419	0.799; 0.008
**USG**	1.013 ± 0.007	1.008 ± 0.004	1.014 ± 0.007	1.007 ± 0.004	0.866; 0.003	**<0.001**; 0.854	0.809; 0.007

Data are shown as mean ± SD (*n* = 10). Body mass and urine specific gravity (USG) were assessed before and after each 15-km time trial. ANOVA results are presented as P value; ηp^2. Pre TT = before time trial; Post TT = after time trial; HSES = high-sodium multi-electrolyte supplementation; PLA = placebo; ηp^2 = partial eta squared. Bolded *P* values indicate statistical significance.

Table S4 | Thermoregulatory Responses

Variable	Condition	Timepoint						2 × 6 RM ANOVA *P-Value*; η2p
		BL	IBE	5 km	10 km	15 km	10PE	
**Tc (Estimated)**	HSES	37.1 ± 0.2	37.1 ± 0.3	38.3 ± 0.4	39.1 ± 0.4	39.4 ± 0.3*	39.3 ± 0.4*	Time, ***P* < 0.001**; 0.968 Condition, ***P* = 0.033**; 0.413 Interaction, ***P* < 0.001**; 0.414
	PLA	37.0 ± 0.1	37.1 ± 0.3	38.4 ± 0.3	39.1 ± 0.3	39.2 ± 0.4	38.8 ± 0.6	

**Tsk**	HSES	31.7 ± 1.6	34.5 ± 0.6	34.6 ± 1.3	34.4 ± 1.3	34.3 ± 1.3	33.6 ± 1.2	Time, ***P* < 0.001**; 0.756<br>Condition, *P* = 0.766; 0.010<br>Interaction, *P* = 0.506; 0.053
	PLA	31.3 ± 1.4	34.4 ± 0.8	34.8 ± 0.9	34.3 ± 1.4	34.2 ± 1.4	33.7 ± 1.5	

Data are shown as mean ± SD (n = 10). Estimated core temperature (Tc) and skin temperature (Tsk) were assessed across six timepoints and are reported in °C. BL = baseline; IBE = immediately before exercise; 5km = 5 kilometers; 10km = 10 kilometers; 15km = 15 kilometers; 10PE = 10 minutes post-exercise; HSES = high-sodium multi-electrolyte supplementation; PLA = placebo. ηp^2 = partial eta squared. * = $p < 0.05$ between conditions at the indicated timepoint. Boldface values denote a significant effect ($P < 0.05$).

Table S5 | Blood Pressure Responses

Variable	Condition	Timepoint			*2 × 3 RM ANOVA P-Value*; **η2p**		
		BL	IBE	10PE	Condition	Time	Interaction
**Systolic (mmHg)**	HSES	130.1 ± 8.9	130.4 ± 6.6	118.5 ± 8.7	0.378; 0.087	**0.002**; 0.512	0.947; 0.006
	PLA	131.3 ± 9.9	132.0 ± 9.9	121.1 ± 11.5			
**Diastolic (mmHg)**	HSES	82.7 ± 5.5	84.4 ± 5.5	78.1 ± 8.2	0.960; 0.000	**0.008**; 0.413	0.847; 0.018
	PLA	81.9 ± 7.7	84.1 ± 4.6	79.0 ± 6.3			

Data are shown as mean ± SD (n = 10). Systolic and diastolic blood pressure were assessed at baseline, immediately before exercise, and 10 min post-exercise. BL = baseline; IBE = immediately before exercise; 10PE = 10 min post-exercise; HSES = high-sodium multi-electrolyte supplementation; PLA = placebo; ηp^2 = partial eta squared. Boldface values denote a significant effect ($P < 0.05$)
